# Characteristics of Internet Addiction/Pathological Internet Use in U.S. University Students: A Qualitative-Method Investigation

**DOI:** 10.1371/journal.pone.0117372

**Published:** 2015-02-03

**Authors:** Wen Li, Jennifer E. O’Brien, Susan M. Snyder, Matthew O. Howard

**Affiliations:** School of Social Work, University of North Carolina at Chapel Hill, Chapel Hill, North Carolina, United States of America; University of Ariel, ISRAEL

## Abstract

Studies have identified high rates and severe consequences of Internet Addiction/Pathological Internet Use (IA/PIU) in university students. However, most research concerning IA/PIU in U.S. university students has been conducted within a quantitative research paradigm, and frequently fails to contextualize the problem of IA/PIU. To address this gap, we conducted an exploratory qualitative study using the focus group approach and examined 27 U.S. university students who self-identified as intensive Internet users, spent more than 25 hours/week on the Internet for non-school or non-work-related activities and who reported Internet-associated health and/or psychosocial problems. Students completed two IA/PIU measures (Young’s Diagnostic Questionnaire and the Compulsive Internet Use Scale) and participated in focus groups exploring the natural history of their Internet use; preferred online activities; emotional, interpersonal, and situational triggers for intensive Internet use; and health and/or psychosocial consequences of their Internet overuse. Students’ self-reports of Internet overuse problems were consistent with results of standardized measures. Students first accessed the Internet at an average age of 9 (SD = 2.7), and first had a problem with Internet overuse at an average age of 16 (SD = 4.3). Sadness and depression, boredom, and stress were common triggers of intensive Internet use. Social media use was nearly universal and pervasive in participants’ lives. Sleep deprivation, academic under-achievement, failure to exercise and to engage in face-to-face social activities, negative affective states, and decreased ability to concentrate were frequently reported consequences of intensive Internet use/Internet overuse. IA/PIU may be an underappreciated problem among U.S. university students and warrants additional research.

## Introduction

Each generation is more familiar with and more reliant upon the Internet. The number of U.S. Internet users increased 257% between 2000 and 2012 [1]. In 2012, the Pew Research Center’s Internet & American Life Survey indicated that approximately 90% of U.S. youth and young adults between 12 and 30 years of age had accessed the Internet [2]. University students are much more likely than the general population to use the Internet: Nearly 100% of U.S. university students accessed the Internet in 2010 [3]. Widespread Internet availability can substantially benefit people by enhancing their access to a broad range of information and creates an avenue for social communication and entertainment [4, 5]. However, the Internet’s penetration into daily life is a serious problem for an increasing number of people, rising to the level of Pathological Internet use (PIU) or Internet addiction (IA), and carrying negative consequences similar to those of other behavioral addictions [6–9].

### Conceptualization of IA/PIU

As Internet use has proliferated, so too have reports of IA/PIU. In the rapidly burgeoning literature in this area, different terms are used to refer to seriously dysfunctional patterns of excessive Internet use. At its most extreme, problem Internet use has been termed “Internet Addiction” or “Internet Dependency”, defined as the “inability to control one’s use of the Internet which leads to negative consequences in daily life [10, 11].” This definition emphasizes the ways in which signs and symptoms of IA are parallel to substance use disorders and pathological gambling disorder. Specifically, symptoms of IA include: a) preoccupation with Internet activities; b) increasing tolerance; c) development of psychological dependency and withdrawal symptoms; d) inability to reduce Internet use; e) Internet use to cope with negative moods and reduce stress; and f) replacing other activities and relationships with recurrent Internet use despite awareness of the deleterious consequences [9, 10].

Other theorists conceptualize these symptoms differently. For these theorists, symptoms associated with Internet-related problems are labeled “Compulsive Internet Use.” Compulsive Internet use is conceptualized as more similar to obsessive-compulsive disorder than addiction [12]. Still other theorists recognize a continuum of less severe Internet-related problems, often referred to collectively as “Pathological Internet Use” or “Problematic Internet Use.” For these theorists, PIU is conceptualized using cognitive and behavioral theories, and defined as a maladaptive coping mechanism for stress and psychological distress, resulting in adverse effects on psychosocial functioning [13–15].

### Instruments that Assess and Diagnose IA/PIU

A variety of instruments have been developed that purport to assess IA/PIU based on different conceptual frameworks. Many of these rating scales, questionnaires, and diagnostic criteria were adopted from DSM-IV-TR diagnostic criteria for substance dependence and pathological gambling disorder [16]. Examples of such measures are Young’s Diagnostic Questionnaire [10, 17], the Clinical Symptoms of Internet Dependency Scale [11], and the Internet Addiction Diagnostic Criteria [18]. Other instruments were developed using cognitive and behavioral models and assess Internet-related cognitions and social functions. Examples of these measures include the Generalized Problematic Internet Use Scale [19] and Online Cognition Scale [20]. Internet addiction is not currently recognized as a formal clinical diagnosis in DSM-5; however, new diagnostic criteria for Internet Gaming Disorder (a subtype of Internet addiction) have been incorporated into Section III of DSM-5 [21], which includes provisional categories of psychiatric disorders requiring further research.

The facets of IA/PIU assessed across these measures largely overlap with different chemical dependency diagnostic criteria, such as salience (i.e., anticipation of and cognitive preoccupation with Internet use), tolerance (i.e., increasing amounts of time spent on the Internet to achieve a same level of satisfaction), withdrawal symptoms, lack of control, and use of the Internet to regulate mood [22]. However, motivations and triggers for problematic Internet use, and craving for Internet use are rarely examined [22]. Further, those instruments often employ unvalidated cut-off points for diagnosing IA/PIU, and therefore it is unclear how to clinically distinguish problematic Internet users from normal users.

### Prevalence of IA/PIU

Greater exposure to the Internet may increase the likelihood of pathological Internet use and the incidence of Internet addiction. As many as 6% to 11% of Internet users in the U.S. are estimated to have IA/PIU [7]. Students may be at substantial risk for the development of IA/PIU problems given the explosive growth of Internet use among youth in the U.S. over the past decade [6]. The accessibility of the Internet on university campuses, the personal freedom and a significant amount of unstructured time, and the academic/social challenges many students experience as they leave home for the first time all contribute to increased rates of IA/PIU [8, 23].

Recent epidemiological studies indicate that IA/PIU affects approximately 1.2% to 26.3% of U.S. university students [24–31]. The majority of the previous studies recruited the samples from a single university campus. A few studies recruited samples from multiple universities by distributing the study information via the university email lists or social media. Three studies evaluated IA/PIU based on DSM-IV criteria for substance use and found the prevalence rates of IA/PIU among U.S. university students were 1.2% to 26.3% [11, 25, 28]. Other studies indicate that 4% to 12% of U.S. university students meet criteria for IA/PIU using the Internet Addiction Test [24, 29, 30]. One study found 8.1% of U.S. college students met criteria for pathological Internet use using the Pathological Use Scale [31]. Moreno et al.’s systematic review of studies reporting IA/PIU prevalence rates for U.S. university students found that 6 of 8 studies reported estimates greater than 8% [27]. Literature also suggests that the prevalence of IA/PIU among the U.S. student population is consistent with similar reports from China, Greece, Britain, and Turkey [32–35].

### Correlates and Negative Consequences of IA/PIU

An extensive international literature has accrued documenting correlates and negative physical and psychosocial consequences associated with IA/PIU. Individuals who suffer from IA/PIU evidence more physical health issues, such as overweight and obesity due to lack of physical activity and sleep disorders [36, 37]; mental health issues, including depressive symptoms, somatic and social anxiety, and attention deficit-hyperactivity disorder (ADHD) [38–41]; temperamental traits such as impulsivity and sensation-seeking [42, 43]; neurological impairments [44, 45]; behavioral problems, including substance misuse, self-injurious behavior, and suicidal ideation and attempts [46, 47]; poorer school and work performance [29]; and more problems with interpersonal relationships compared to their counterparts without IA/PIU [48].

The burgeoning literature indicates that many university students suffer from a variety of health and psychosocial problems due to IA/PIU. However, the majority of research concerning IA/PIU in U.S. university students has been conducted within a quantitative research paradigm. Though quantitative studies offer important clinical and research implications, they frequently fail to contextualize the problem of IA/PIU. Without this contextualization, specific clinical presentations, including triggers for and patterns of use, have gone unidentified. In addition, it is unclear from these quantitative studies which physical and psychosocial consequences individuals find the most adverse and therefore would be the most beneficial to target during treatment.

### Current Study

In order to address this critical gap, our research team conducted an exploratory qualitative study to investigate a range of issues relating to IA/PIU including the natural history of IA/PIU problems; common affective, interpersonal, and situational triggers of intensive Internet use; preferred patterns of Internet activity; and adverse psychiatric, psychosocial, and health consequences of intensive Internet use. Findings of this qualitative research will provide a more detailed picture about IA/PIU in university students that may help us to contextualize the results from previous quantitative research and discover all relevant IA/PIU-related experiences in U.S. university students.

## Methods

We employed exploratory qualitative methods including four focus groups to obtain detailed descriptions of IA/PIU from 27 university students. Participant recruitment for the focus groups was conducted between March and April, 2012. Participants were allocated to one of four focus groups based on their availability. Ultimately each focus group consisted of 6–8 participants, and lasted approximately one hour. Descriptive data were collected during focus groups to describe participants’ sociodemographic and Internet usage characteristics.

Focus groups are guided group discussions on one or more topics with participants who share similar experiences and/or who possess information and knowledge about the topics of discussion [49]. We employed focus group methods in this study because: a) the target population, university students who self-identify as Internet over-users, could directly provide insights and knowledge regarding their intensive Internet use; and b) group dialogue tends to generate rich information as group discussions inspire participants to share personal experiences and perspectives in a way that teases out the nuances and tensions of complex topics [50].

### Focus Group Materials and Measures

Focus group assessment materials consisted of 22 open-ended questions and a set of objective measurement instruments (S1 Document). The group discussion was semi-structured, with the facilitator asking a series of open-ended questions. The group discussion guide was developed and refined by the investigators based on the research aims, relevant substantive theories, and pilot testing. Major issues explored in the focus groups concerned a) participants’ Internet use experiences, such as the online activities they devoted the most time to, reasons they enjoyed those activities, average amount of time they spent on the Internet daily, and longest period of time they spent on the Internet in one continuous session of use; b) affective, interpersonal and situational factors triggering intensive Internet use; and c) negative consequences of Internet overuse, including adverse effects on physical, mental, social, and professional well-being. We conducted in-depth individual interviews with six university students to pilot test the questions we used subsequently for the focus groups.

Young’s Diagnostic Questionnaire (YDQ) [10] and the Compulsive Internet Use Scale (CIUS) [51] were employed to assess IA/PIU and validate students’ self-identification as problem Internet users. We chose the YDQ because it is a short questionnaire and widely used in the extant literature examining the prevalence and correlates of IA/PIU among youth and young adults (Li et al., 2014). Using the same measure as these previous studies enabled us to compare our findings to those in the published literature. Our team chose to pair the YDQ with the CIUS because the CIUS is designed to measure similar constructs to the YDQ; however, the CIUS demonstrates superior psychometric properties [51]. The benefit of using two standardized measures, in part, is to strengthen the validity of the results through data triangulation. The YDQ and the CIUS have been widely employed to investigate the prevalence and correlates of IA/PIU. However, there are no valid cutoff points to make any clinical diagnosis regarding IA/PIU using these measures. Therefore, no diagnoses were made in this study.

The YDQ is adopted from the DSM-IV-TR criteria for pathological gambling disorder, consisting of 8 questions that assess signs and symptoms of IA/PIU including preoccupation, salience, tolerance, withdrawal symptoms, and impairment of psychosocial functioning [10]. Participants answering “yes” to 5 or more questions were identified as having IA whereas these meeting 3 or 4 criteria were considered to have “sub-threshold IA” [52]. The internal consistency reliability of the YDQ in this study was .69.

The CIUS includes 14 items rated on a 5-point Likert-type scale, ranging from 0 (never) to 4 (very often). The CIUS assesses severity of compulsive/addictive Internet use behavior, including loss of control, preoccupation, salience, conflict, withdrawal symptoms, and Internet use for purposes of coping with problems and dysphoric moods. Higher scores indicate greater severity of compulsive Internet use. The CIUS has an internal consistency reliability of approximately .90 [51]. In this study, the CIUS had an α = .92. Guertler and colleagues have recommended use of a cutoff score of ≥ 21 for the estimation of problematic Internet use [53].

### Ethics Statement

This study was approved by the University of North Carolina-Chapel Hill Institutional Review Board and performed in accordance with the Declaration of Helsinki. Written consent was obtained from all participants before the focus groups commenced.

### Participants

Our team used a purposive sampling strategy by recruiting participants who were graduate or undergraduate students enrolled at a large public university in the southeastern United States. Purposive sampling was chosen with the following goals in mind: to generate information-rich data about Internet use among students who self-identify as intensive Internet users, to identify triggers of Internet use among intensive Internet users, and to explore both physical and psychosocial consequences of intensive Internet use.

A recruitment email was distributed via the university listserv. The university listserv includes all undergraduate and graduate students, exchange students, and recent alumni (graduated within the past 2 years). In the email the research team introduced the purpose of the study, study participation requirements, and identified the research team as social workers working for the School of Social Work. Participants responding to the recruitment email who were current graduate or undergraduate students enrolled at the university, self-identified as intensive Internet users, who reportedly spent ≥ 25 hours/week on the Internet for non-school or non-work-related purposes, and who experienced one or more physical and/or psychosocial problems caused by intensive Internet use were eligible for study participation. Physical and/or psychosocial problems were intentionally assigned a very low threshold for inclusion (i.e., report of any lifetime problem that the participant attributed to their Internet use) to elicit wide variation in experiences with Internet use.

More than 30 students responded to the email within two hours of the study solicitation and expressed a willingness to participate in the study. Several students revealed that they used the Internet > 40 hours per week for non-school or non-work-related reasons, and suffered multiple physical and psychological problems due to intensive Internet use. By responding to the initial recruitment email, thirty-nine students agreed to participate in the focus groups. The research team responded via email to schedule a focus group time with all 39 respondents, and confirmed that time with a second email. Twelve students failed to attend their scheduled groups for unknown reasons. Thus, four groups were held including 27 students. Participants were assigned to one of four group sessions based on their availability. Sample characteristics are reported in Table 1. The average age of participants was 21 (SD = 3.6), ranging from 18 to 36. A majority (63.0%, N = 17) of students were women and the sample was racially diverse. As shown in Table 1, participants represented 11 majors in the university, and 72.5% (N = 20) were undergraduates.

**Table 1:** Characteristics of 27 University Students Who Self-Reported Intensive Internet Use.

Variables	% (N)	Mean (SD)
Age		21.0 (3.6)
Gender		
Male	37.0% (10)	
Female	63.0% (17)	
Race		
White	25.9% (7)	
Black	33.3% (9)	
Asian	33.3% (9)	
Latina/Latino	7.4% (2)	
Student status		
Undergraduate	81.5% (22)	
Graduate	18.5% (5)	
Major		
Biology	7.4% (2)	
Business/Economics	11.1% (3)	
Information science	3.7% (1)	
Journalism/Communication	18.5% (5)	
Mathematics	3.7% (1)	
Medicine	7.4% (2)	
Nutrition	3.7% (1)	
Philosophy	3.7% (1)	
Political science	11.1% (3)	
Psychology	7.4% (2)	
Public policy	7.4% (2)	
Sociology	7.4% (2)	
Undecided	7.4% (2)	

### Data Collection

Four focus groups were conducted in a conference room on campus. Each focus group lasted approximately one hour. The number of participants attending each group ranged from 6 to 8, to assure that a wide range of ideas and opinions were represented. The last author facilitated all focus groups. The first author accompanied the last author and was responsible for taking notes during each focus group. The notes supplemented transcription data by capturing changes in participants’ “body language” or other nonverbal communication. The presence of multiple observers at group sessions allowed for observer triangulation to improve the reliability and validity of findings emerging from group discussions [54]. Prior to each focus group, participants completed the YDQ, the CIUS, and a brief sociodemographic survey. During the focus groups, participants answered questions related to their Internet use experiences and perception of the severity of their own problematic Internet use.

### Data Analysis

Audiotapes of focus group sessions were transcribed verbatim, and checked for accuracy by all authors. No software was used to assist in the coding or transcription of data. Three analysts organized codes into umbrella codes and subcodes (i.e., a code tree). First, codes were generated from the research aims and published reports that guided the research (e.g., research findings pertaining to correlates and consequences of IA/PIU). Then, we reviewed and revised the theory-driven codes in context, providing codes with labels and definitions reflective of the raw data. Further, in accordance with DeCuir-Gunby et al.’s recommendations [55], the second round of coding was conducted on the level of meaning via a data-driven method, enabling codes to be developed on the sentence and paragraph levels. In this round of coding, we investigated and identified any new themes and divergent perspectives that emerged from the data that had not been captured by the theory-driven codes, and determined if the theory-driven codes needed to be expanded or a new code needed to be developed.

Each of the study investigators independently reviewed and coded focus group transcriptions using the given framework to enhance the reliability and validity of study findings through analytical triangulation [54]. Coding discrepancies among authors were resolved through mutual discussion and agreement. Patterns were identified and categorized together by all investigators, until the analysis showed convergence and saturation. Methods to enhance the rigor of the research included implementing data triangulation by using more than one method to collect similar data (e.g., using two separate self-report measures, demographic questionnaires on past use). In addition, regular debriefing and consultation among research team members aided in clear functional definitions of all codes and negative case analysis [54].

## Results

### Descriptive Results

Participants described their current patterns of Internet use with respect to the daily amount of time they spent on the Internet and longest period they ever spent on the Internet in one continuous session of use. The amount of time students reported spending on the Internet daily ranged from 5 hours to “all day,” due to the widespread use of mobile devices (e.g., smartphones and tablet computers) with data coverage (e.g., “I feel like I’m on the phone all the time constantly checking”). Many participants noted they could not accurately differentiate the amount of the time spent on the Internet for school work or work-related purposes from that for non-school/non-work-related purposes (e.g., “If I’m writing a paper, then I’ve got my browser open, or I’m on my phone”). The longest period of time participants reported spending on the Internet in one continuous session ranged from 3 hours to all day (e.g., “Once its summer, I’ll be on it [the Internet], like, an entire day”). During those sessions, participants described engaging in different activities including online shopping, video watching, and website browsing. Other participants described use of a specific application for a long period of time, including playing video games and watching videos (e.g., TV shows and movies) on the Internet.

The age at which participants reported they first accessed the Internet ranged from 6 to 19, with an average age of 9 (SD = 2.7). The age at which participants reported they first thought they had a problem with Internet overuse ranged from 10 to 32, with an average age at onset of problems of 16 (SD = 4.3). Table 2 reports characteristics of participants’ self-reported IA/PIU.

**Table 2:** Internet Use Characteristics of 27 Participants Who Self-Reported Problem Internet Use.

Variables	% (N)	Mean (SD)
Age first accessed the Internet		9.3 (2.7)
Age first recognized having a problem with Internet use		16.2 (4.3)
Total YDQ Score		4.7 (2.0)
YDQ ≥ 5	48.1% (13)	
YDQ = 3 or 4	40.7% (11)	
CIUS Total Score		33.3 (11.2)
CIUS ≥ 21	96.3% (26)	

YDQ ≥ 5 indicates Internet addiction. YDQ scores of 3 or 4 = potential IA. CIUS ≥ 21 indicates compulsive Internet use.

Almost half (48.1%, N = 13) of the student sample scored five or more on Young’s Diagnostic Questionnaire (YDQ), and therefore scored above the suggested cut-off point for IA. Another 40.7% (N = 11) scored a three or four on the YDQ, reflecting the suggested cut off for sub-threshold IA. Virtually the entire sample exceeded the recommended cutoff for compulsive Internet use according to the Compulsive Internet Use Scale (CIUS). More than half (63.0%, N = 17) of students reported using the Internet to escape from problems or relieve a negative mood. As for negative consequences of intensive Internet use, 63.0% (N = 17) of students reported sleep deprivation; 44.4% (N = 12) reported they neglected school work and other daily obligations due to their intensive Internet use. The correlation between the YDQ and the CIUS was .79.

### Qualitative Results

**Fig 1:**
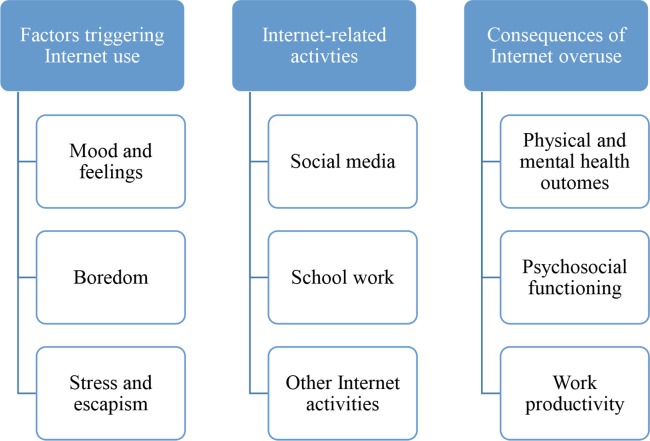
Diagram of Qualitative Themes and Subthemes.

Three overarching themes emerged from the focus groups relating to: a) factors triggering Internet use for non-school or non-work-related purposes, b) Internet-related activities, and c) consequences of Internet overuse. Fig. 1 shows a diagram with all qualitative themes and subthemes, please see Fig. 1. In order to contextualize quotes, focus group participants’ gender and race are provided. For the ease of the reader, pseudonyms have been given to participants, so that quotes given by the same individual are identifiable.

**Theme 1: Factors triggering Internet use**. This theme was characterized by the emotional, interpersonal, and situational factors that heighten college students’ desire to use the Internet for non-school/non-work-related purposes. Subthemes included: a) mood and feelings, b) boredom, and c) stress and escapism. Many participants noted that more than one of these factors contributed to their Internet overuse at different times.

For several participants, Internet overuse was triggered by strong feelings and moods. For some, the strongest urges came with positive emotions (e.g., “When I am crazy happy, I want to let my friends know. I feel that I want to post it on Facebook” [“Andrew”, a white man]). For others, negative emotions were a bigger trigger (e.g., “If I have a bad day then I deserve a reward kind of…” [“Lily”, an Asian woman]). Regardless of the valence of the emotion, most participants noted that particular feelings and moods triggered desires to engage in specific Internet activities. “Nancy,” an Asian woman described her desire to use a particular Internet application as a coping mechanism for sadness:

If I’m really depressed, I won’t get on Facebook, I don't want to talk to anyone. I won’t use anything like a social networking kind of thing, but I’ll definitely go on something like Tumblr to look at funny things for like an hour.

Other students found they used social media more at times of interpersonal conflict as a way of managing their anxiety about the conflict. While some participants reported “updating my status constantly,” others reported checking other’s status. “Jessie,” an African American woman, noted:

If I ever have a fight with somebody, or tension, or drama …I’ll just get on Facebook to see if they said anything about their mood, or anything about me in particular, or something like that.

Furthermore, participants had differing desires for use based on mood, with some having more awareness of these patterns than others. “Alice,” an Asian woman, discussed her own patterns of use since entering college, stating:

I found out I go online more when I am sad than happy. When I am sad, I just want to talk to a friend from overseas via long distance calls or something. So I just chat with them online. And when I am happy, I don’t usually go online.

Many participants reported that boredom triggered their desire to use the Internet. Students discussed the Internet as their primary strategy for coping with boredom. “Tom,” a white man, described his experience this way: “If I get bored, that’s the first thing I go to.” Others seemed to link the Internet to specific types of boredom relief (e.g., laughing, connecting with others, and information retrieval). “Mike,” an African American man stated: “Whenever I feel bored, and if I feel stressed, I just get on the Internet to relax, maybe have a laugh or two.” For participants, including “Mike,” the Internet was a means of relief any time boredom arose due to easy access on mobile devices with data coverage: “I think when you get bored, you always want to log on to that thing; like riding the bus to the class, you feel bored, you don’t have friends, you just get on because you’re bored.”

In addition to moods, feelings, and boredom, school and interpersonal stressors triggered students’ desire to use the Internet. “Sue,” an Asian woman, reported a desire to “avoid things, so I get on the Internet. You don’t have to think about anything. You just watch and take it in.” For some, the Internet was a time-limited break:

I think for me, like when I’m really stressed about school, when I need a break, or have a problem I usually go to the computer to get away from school, get away from the problem for an hour or two [“Jessie,” an African American woman].

For others, time spent on the Internet was more difficult to control and ended up adding to their initial stress:

I’m like, if I’ve been on the Internet for 8 hours, and I haven’t done anything, I get stressed out and I tell myself like “how could you do this, waste this much time?” I get annoyed with myself, but then because I’m annoyed, I will look for something funny to laugh at [“Sue,” an Asian woman].

Some participants noted the desire to escape obligations as a trigger for Internet use. “Sarah,” an Asian woman, described that desire in this way: “For me, like procrastination, I don’t want to do anything else, so I just, sometimes I just want to be entertained. I don’t want to do my homework.”

**Theme 2: Internet-related activities**. This theme describes the online activities participants’ favored and the reasons for their enjoyment of those activities. Many participants engaged in multiple activities while on the Internet. Subthemes included: a) social media, b) school work, and c) other Internet activities.

Most participants reported using some form of social media. Social media includes applications like Facebook, Twitter, Pinterest, and Tumblr. Due to the accessibility of social media sites on mobile devices, many participants noted their use as part of their daily routine (e.g., “If I’m not sleeping, then I’m on Twitter or Facebook on my phone…all day long” [“Lydia,” an African American woman]). The extent of daily use ranged from casual (e.g., “For me, I like sharing thoughts or ideas or moods with followers on Twitter or Facebook. Like when you think of something, you’re like ‘Oh, I’ll tweet that’” [“Jessie,” an African American woman]) to compulsive (e.g., “It becomes a habit that when I wake up in the morning, the first thing I do is to check Facebook, like repeatedly. If you don’t do it, you’ll feel like you’re missing something” [“Sue,” an Asian woman]). The emergence of multiple social media sites gives users a variety of channels to connect with their peers. Some participants described use of multiple social media sites. “Sharon,” an African American woman, described her use this way:

Most of the time I like to refresh my news feed on Facebook, or look at my followers on Twitter to see what everyone is talking about, and [if] people post a dramatic status [on Twitter], then I’ll go to look at their [Facebook] profile links and see what they’ve posted.

Other participants like “Christian,” an African American woman, reported very intensive use of one site:

There are days that I’ve tweeted 100 times …I’ll get up and check Twitter, or when I get on the bus to class, I’ll check Twitter, or in class, I’ll check Twitter, and during lunch, I’ll check Twitter, and before I go to sleep I’ll check Twitter.

Although some participants highlighted the importance of social media in their everyday lives, many were quick to point out the practical, work-related functions that the Internet fulfills. As “Christian,” an African American woman, astutely observed: “The Internet is not only Facebook and Twitter and Pinterest but also email, and Google, and the library database on the Internet.” In fact, many students reported that professors required students to use the Internet in completing their assigned class work, including writing blogs, taking online classes, and accessing virtual class materials. “Matt,” an Asian man, was very positive regarding the importance of the Internet to his education, stating, “My research requires specific information that the Internet provides pretty conveniently. For me, the quality of life is increased.” Other participants were ambivalent, stating that accessing school work/work-related materials on the Internet were both a help and a hindrance. “Christian,” an African American woman, noted: “You’re on Facebook, and Google, and your email, and Twitter, and you’re writing a paper, and you’re reading something. It’s just like moving constantly.” Universally, participants acknowledged the convenience and necessity of the Internet as a part of the collegiate environment. “Kate,” a white woman, stated: “I frequently use the Internet mostly for class and clarification of topics. Cut out the Internet completely, I don’t know how to survive in a university setting.”

The final subtheme, “other Internet activities,” included recreational activities such as watching video streams, playing online video games, browsing entertainment, social networking, and news websites, posting on forums (e.g., Reddit), and general searches. These activities were generally performed in conjunction with work and/or social media. “Angela,” an African American woman, reported “I listen to music on the Internet while I’m doing my homework, cleaning my room, or playing *Zelda* (a video game), or watching others online playing *Zelda* at the same time.” Other participants engaged in only one activity at a time, saying they preferred certain activities to others. Examples include news retrieval (“I think my chief source of news is on the Internet. I read 3 or 4 newspapers on my feed, and that is very important” [“Matt,” an Asian man]), online gaming (“I play with random people on the Internet, and have interactions with them, like when I play basketball games. You kind of just play them, and play them” [“Tom,” a white man]), and video streaming (“For me, spending more time watching movies and shows, than actually doing social media. This changes over time, from watching movies to doing something else” [“Matt,” an Asian man]). “Claire,” a white woman, reported that online shopping was particularly appealing, stating “I hate going to the mall, and hate trying on clothes, now I don’t have to. It’s just right there online.” Regardless of the activity, the subtheme “other Internet activities” highlights the widespread utility and appeal of the Internet, but also underscores the risk for potentially problematic Internet use.

Whether students are using the Internet to enhance interpersonal connections and social networking, school work, or recreation, the Internet offers a variety of readily available options encouraging constant use. In fact, students noted that peers and professors facilitate and reinforce their Internet use, which therefore could be a potential risk for those who are more at risk of developing IA/PIU. “Kate,” a white woman, described the expectations of others this way: “For checking my email, it’s like I don't get joy from it, feels like I have to, I have to respond when someone at work emailed me, or I don’t know if I should have to.”

**Theme 3: Consequences of Internet overuse**. The theme “consequences of Internet overuse” was characterized by participant descriptions of short- and long-term effects of Internet use. Subthemes included physical and mental health outcomes, psychosocial functioning, and work productivity. Though not all effects were negative, participants were more apt to point out negative consequences, particularly with regard to health and work.

Participants discussed adverse health consequences as an effect of Internet overuse. A few participants reported general concerns regarding physical health. These concerns included sleep deprivation (e.g., “I think lack of sleep. I know even when I’m done with work, it’s like 12 or 1. I’ll be up ‘till like 3 because I’m doing some random stuff on the Internet” [“Nancy,” an Asian woman]), lack of exercise (e.g., “I’ll plan to exercise, like I’ll sit there, keep reading stuff, and like ‘too bad I didn’t get to exercise’ [“Kevin,” a white man]), and poor posture (e.g., “…our generation has pretty bad posture due to typing a lot and sitting” [“Mike,” an African American man]). “Tom,” a white man, pointed out the intersection between mental and physical health, stating “I get down on myself, feel frustrated if I spend a whole lot time on the Internet one day, instead of doing something physical or go outside.”

Other students focused mostly on their experience of psychological symptoms. For some participants, anger and frustration were the most prevalent symptoms. “Heather,” an African American woman, reported: “The first thing of the day is to get on Facebook or Twitter. If I hear something stupid, it will annoy me the rest of the day.” Similarly “Lucy,” an Asian woman, noted a difference in her day-to-day irritability:

I think it makes me feel like blah after being on the Internet a long time, just like I feel like I wasted a lot of time. I think even sometimes I don’t have social interactions with people during long periods of the day, I got more irritable.

Other participants reported experiencing sadness and depression after Internet use. For some, this sadness was sparked by comparing their current lifestyle to that which their peers had posted on social media. “Andrew,” a white man, elaborated by stating:

Usually most people post the best part of their life actually, so half the time you’re going there, and just see like “Oh, I am having so much fun, and I am at the beach, partying with hot girls.” And you are like “I’m in my dorm room, and I’m… I’m working at McDonald’s.” I doubt… their life is… much better than mine. But when I’m already depressed, and get on the Internet and see that, I’m like “Yeah man, I suck.”

Student’s use of the Internet and subsequent health reports may be related to the specific Internet activities they are engaged in and to their patterns of Internet use. As “Heather,” an African American woman, pointed out: “If you’re a social person, then it [social media] adds to it. It’s like a faster outlet…But if you’re not, then instead you’re just watching.” Quotes such as this one highlight the dual or paradoxical effects of the Internet on social functioning. That is, the Internet can enhance a student’s social life; however, when used excessively and in ways that foster and reinforce social isolation, its use can diminish the quantity and quality of face-to-face social interactions. Some participants complained that their face-to-face interactions were hindered by their peer’s use of the Internet. “Nancy,” an Asian woman, explained her experiences this way:

I have this thing, like especially when I’m eating with someone, they pull out their phone and they start to check their Facebook, Twitter, or something like that, I’ll look at them and I’ll be like “really, you’re going to do it in front of me right now?”

“Den,” an African American man, noted that reliance on the Internet for social interactions may lead to a lack of face-to-face communication skills: “When you’re behind the computer, you spend time making the perfect message… But when you’re face to face, [the person] is kind of socially awkward, not really there.” Furthermore, in a quote echoing the sentiments of many, “Lydia,” an African American woman, emphasized that Internet overuse negatively affected her relationship quality, stating: “I would go home, instead of talking to my aunt and cousins, I just sat on the couch, playing with my laptop or my phone. Don’t really socialize with anybody else. So I don’t really talk with anyone.”

Conversely, other participants noted positive social effects of Internet use. The Internet can facilitate connections to family, friends, and community supports. “Fred,” an African American man from focus group two, explained it this way:

I felt like if you’re on Twitter, you’re like connected. If you’re on campus, everyone is close. But at the same time, Twitter makes it closer…I feel like you let people know what you’re doing more publicly, so they can hang out with you if you want to.

The Internet appeared to be especially important for participants in long distance relationships. “Angela,” an African American woman, described the benefits of using the Internet to keep up with family that lived far away, stating: “I think it’s helpful. There are a lot of family members that I haven’t really talked to…So I can just send a quick email and say ‘hey, how are you,’ instead of calling them.”

Academic productivity, the final subtheme, describes how participants’ perceived the effects of Internet use on overall school work and productivity. Many participants noted the negative effects of Internet overuse on their general academic performance. “Lydia,” an African American woman, stated: “I feel like that if not for my Internet use, my grades could be 10 times better.” Some participants, like “Jessie,” an African American woman, related this to an inability to focus: “My ability to concentrate on one thing for a long time is seriously impaired… I can’t even focus for 2 minutes.” Other students noted that their work quality suffered due to procrastinating on the Internet. “Nancy,” an Asian woman, reported: “My school work suffered a lot from Internet use…being on the Internet is like you’re procrastinating so much, eventually you get to the point that ‘I need to get this done…’ You’re just not all the way there.” Generally, students reported that while the Internet was necessary for school, the consequences of Internet overuse were antithetical to their school goals.

## Discussion

This study investigated a range of issues relating to IA/PIU in U.S. university students including the natural history of Internet overuse problems; common affective, interpersonal, and situational triggers of intensive Internet use; preferred patterns of Internet activity; and adverse psychiatric, psychosocial, and health consequences of intensive Internet use. This study did not attempt to determine the prevalence rate of Internet addiction in U.S. university students. Instead, we intended to provide rich and detailed descriptions of students’ experiences with intensive Internet use/Internet overuse by directly quoting participants’ words in the focus groups. Further, the qualitative themes that were generated from focus group discussions contextualized relevant findings from previous quantitative studies.

Many students acknowledged that it was difficult to accurately calculate the total amount of time they spent on the Internet per day because unlimited data plans on mobile devices (e.g., phones and tablets) mean Internet is constantly available. However, students were still able to self-report consistently and accurately across both self-report qualitative and standardized measures, validating both qualitative and quantitative results. Many students stated they could not accurately differentiate the amount of the time spent on the Internet for school or work-related purposes from that for non-school/work-related purposes. Some studies have suggested a positive association between the total amount of time spent on the Internet and IA/PIU in university students [26, 56]; however, it may be more accurate to differentiate the amount of time spent on the Internet for work and/or school related purposes from the amount of time spent on the Internet for entertainment purposes [29]. For non-school/work related Internet activities, participants engaged minimally in the use of online video games. Social media use was pervasive among the sample. Student’s academic relationship with the Internet is dynamic and varied. Although they note the pervasive and negative consequences of overuse, they also point out the benefits of the Internet in their academic work.

Qualitative findings demonstrated that negative emotions (e.g., depressive mood, sadness, and anger), boredom, and stress associated with social- and work-related obligations were common emotional and situational triggers for many students to engage in intensive Internet use. Unfortunately, use of the Internet as a coping strategy for negative psychological states may also perpetuate these states in the long-term. Research suggests that use of the Internet as a coping mechanism may be similar to self-medication with alcohol and other psychoactive drugs [13]. Theorists have suggested that problem Internet use is a palliative coping mechanism for negative affective states and mental distress [13, 15]. For students in this study, the negative emotional states resulting from palliative Internet use were related to anger and frustration. Reasons for frustration varied (e.g., feeling guilty due to spending lengthy and unproductive time on the Internet, angry with other people’s behavior on the Internet); however, students reported intensive Internet use both contributed to and exacerbated negative emotional states. Many students had the immediate desire to engage in different activities on the Internet (e.g., browsing social media sites) when feeling bored, particularly when the Internet (e.g., laptop computers and mobile devices with Internet access) was readily available. Youth high in boredom susceptibility, impulsivity, and novelty/sensation seeking temperaments are at elevated risk for addictive behaviors [57, 58]; thus, it is intriguing that many students in this study reported Internet use as a primary means of coping with boredom. Studies in international settings have found that youth with IA/PIU shared similar genetic and temperamental traits to individuals suffering from substance use disorders and behavioral addictions, including impulsivity and sensation seeking [7, 9, 42].

Study participants reported a variety of adverse health and psychosocial consequences related to intensive Internet use. Many students failed to exercise and engage in face-to-face social activities because of the excessive amounts of time they spent on the Internet. Prior research has linked Internet use to weight gain and obesity [59] and theorists have speculated that the explosive growth of Internet use among youth and young adults may be a key factor in the epidemic of obesity in the U.S., China, and elsewhere [60]. Many students in this study cited Internet overuse as a key factor in sleep deprivation. This finding is consistent with previous studies that have indicated that students who suffer from IA/PIU were more likely to experience sleep disturbance, lack of sleep, and insomnia [30, 61]. Students’ in this study noted that their decreased sleep was predominantly the result of procrastination on the Internet. Some students had to sacrifice their sleep time to rush through school work due to spending lengthy and unproductive time on the Internet.

Excessive/problem use of social media sites in youth and emerging adults have been examined and documented [62–64]. Many students in this study regarded social media ambivalently, noting that such media can play both a facilitating and inhibiting role vis-à-vis face-to-face socializing, depending on the level and nature of use and individual characteristics of the user. Unlike results of previous studies, which found that university students often meet and socialize with other people in chatrooms in order to cope with symptoms of depression [24, 25, 29], some students in this study noted that when they felt “sad” or “depressed” they preferred to watch videos or browse blogging and/or bulletin board sites (e.g., Reddit) on the Internet. Students reported avoiding socialization with other people on the Internet while experiencing symptoms of depression.

Several quotes from this study indicate that access to the Internet has lowered students’ thresholds for boredom such that individuals become bored more quickly and have increased difficulty concentrating on imperative, school/work-related tasks. Theorists have conjectured that excessive Internet use may affect brain function in ways that reduce the ability to concentrate [65]. Also, previous studies have linked attention-deficit hyperactivity disorder (ADHD) to IA/PIU in Korean university students [41, 66]. Findings from this study indicate these previous findings may not be culturally bound.

Additionally, contrary to much of the reported literature [9], participants in this study engaged minimally in use of online video games. This finding may be due to the composition of our sample, a significant majority of which was comprised of women. Previous studies indicated that men are more likely to play video games excessively and develop problems such as video gaming addiction than women [23, 67]. Cultural factors may also play a role in the lower levels of online video game playing reported in this sample relative to levels identified in studies of East Asian university students [23]. Additionally, video gaming in this sample may be minimally reported due to the way the study purpose was advertised as exploring students’ experiences with Internet overuse. Students might have the expectation that they were primarily to discuss their experiences of using the Internet via computer, instead of playing video games on the Internet via other game consoles (e.g., Xbox 360). Stigmatization of excessive and/or problematic gaming may also minimize reporting in a group setting.

Ultimately, this study created almost as many questions as it answered. Specifically, this study’s findings shed additional light on several previous findings that have been highlighted in the literature as unclear, or otherwise exploratory in nature. For example, almost the entire sample (99.7%, 2 SD from mean) first accessed the Internet prior to entering college (M = 9 years of age, SD = 2.7); and many students didn’t self-identify as having problems associated with intensive Internet use until their late teens/after entering college. Some previous findings have suggested that the number of years using the Internet was associated with IA/PIU [34, 56]; however, other research has not supported such conclusions [26]. Future studies are necessary to clarify whether the early onset of Internet use or Internet overuse can act as a predictor for future IA/PIU.

In addition, this study highlights some of the similarities between IA/PIU and other behavioral addictions. It is truism in the substance abuse and mental health fields that early onset of substance use portends a more problematic course and poorer prognosis than later onset [68]. However, because there are no longitudinal studies that investigate the developmental trajectory of IA/PIU, we cannot draw any conclusion about the long-term trajectories of IA/PIU among these students. Additional study of the natural history of IA/PIU in U.S. university students and associated adverse health and psychosocial consequences would also inform prevention and treatment initiatives and thereby potentially enhance their effectiveness.

As mentioned previously, students in this study spent many hours on social media sites. The amount of time spent on social media sites may suggest habit formation rather than addictive properties, although previous studies have suggested that students found Facebook was addictive [62]. Further research is necessary to determine the addictive components of social media use among university students. In particular, future studies should attend to the presence of withdrawal symptoms when students are unable to use social media sites. Thus, future studies may be necessary to examine the specific activities that students engage in on social media sites (e.g., primarily posting on social media sites vs. primarily browsing other people’s post) and how the different activities effect clinical outcomes of intensive social media use. Research on instrument development that assesses problem social media sites use may benefit from including questions that capture the different nuances. Finally, further studies are necessary to establish clinical diagnostic criteria that can accurately differentiate normal users from students who suffer from IA/PIU. More research is needed to investigate whether these students are amenable to and would benefit from formal prevention and treatment interventions.

Study limitations include the small sample size, single site location of the investigation, and exploratory nature of the findings. These factors all may limit the generalizability of results. The recruitment email that was sent to the entire student body of the university was used as a screening tool; however, it is possible that students self-selected into the study and may have differed from students with IA/PIU problems who declined to respond to the recruitment email. Additionally, the standard measures for IA/PIU used in this study do not have clinical or empirical cutoff scores established for distinguishing between IA/PIU and normal Internet use. Therefore, we are relying on participants’ own self-reflections and self-reports, which are subjective in nature.

Despite these limitations, the university where this research was conducted is not unlike many other large public universities and the study sample was diverse with respect to race and gender. Further, participants’ self-reflections and qualitative responses regarding their own perceived problematic Internet use add depth to findings and help to contextualize previous research results related to IA/PIU in university students, including the natural history of PIU, triggers and patterns of IA/PIU, and consequences of IA/PIU. Many of the students we studied were emphatic about the harms they had suffered due to intensive Internet use/Internet overuse. It is likely that most students at risk for or suffering from IA/PIU problems in the U.S. receive no specific preventative or treatment interventions for their Internet overuse problems. Although a substantial body of international literature has accrued identifying adverse consequences of IA/PIU in university students, university campus health care and other health care agencies struggle to identify IA/PIU in university students and provide treatment due to a lack of clinical diagnosis tools and appropriate interventions [7, 23]. We hope our findings will stimulate further investigation in this emerging area.

## Supporting Information

S1 Document Survey Questions for Sociodemographic and IA/PIU Characteristics, and Focus Group Discussion Guidance. (DOCX)

S1 Table Data Set for Sample Characteristics of 27 Participants Who Self-Reported Intensive Internet Use. (DOCX)

S2 Table Data Set for Young’s Diagnostic Questionnaire (N = 27). (DOCX)

S3 Table Data Set for the Compulsive Internet Use Scale (N = 27). (DOCX)
