# Supplementary material for: Characteristics of Internet Addiction/Pathological Internet Use in U.S. University Students: A Qualitative-Method Investigation
Source: PLoS One. 2015 Feb 3;10(2):e0117372. doi: 10.1371/journal.pone.0117372 (PMC4315426; doi:10.1371/journal.pone.0117372)
Supplement: S1 Table — (DOCX) [file pone.0117372.s002.docx]

**Table S1** Data Set for Sample Characteristics of 27 Participants Who Self-Reported Intensive Internet Use

| Case ID | Age | Gender | Race | Students Status | Major | Age first accessed the Internet | Age first recognized having a problem with Internet use |
| --- | --- | --- | --- | --- | --- | --- | --- |
| 1 | 20 | Female | Asian | Undergraduate | Psychology | 7 | 13 |
| 2 | 22 | Male | White | Graduate | Nutrition | 10 | 20 |
| 3 | 19 | Female | White | Undergraduate | Information science | 8 | 17 |
| 4 | 25 | Female | Asian | Graduate | Medicine | 11.5 | 16 |
| 5 | 20 | Female | Black | Undergraduate | Sociology | 9 | 11 |
| 6 | 20 | Female | Latino | Undergraduate | Journalism | 8 | 18 |
| 7 | 22 | Female | Latino | Undergraduate | Political science | 9 | 18 |
| 8 | 20 | Female | Black | Undergraduate | Political science | 11 | 15 |
| 9 | 20 | Female | Black | Undergraduate | Communication | 8 | 11 |
| 10 | 18 | Male | Black | Undergraduate | Undecided | 8 | 15 |
| 11 | 18 | Male | Asian | Undergraduate | Undecided | 11 | 12 |
| 12 | 20 | Male | White | Undergraduate | Public policy | 6 | 18 |
| 13 | 21 | Female | Asian | Undergraduate | Mathematics | 6 | 10 |
| 14 | 26 | Female | White | Graduate | Journalism/Communication | 12 |  |
| 15 | 18 | Male | Black | Undergraduate | Political science | 8.5 | 18 |
| 16 | 21 | Male | Black | Undergraduate | Philosophy | 10 | 16 |
| 17 | 21 | Female | White | Undergraduate | Public policy | 6 | 17 |
| 18 | 21 | Female | Black | Undergraduate | Biology | 11 | 18 |
| 19 | 22 | Male | White | Undergraduate | Economics | 10 | 19 |
| 20 | 22 | Male | White | Undergraduate | Business administration | 12 | 18 |
| 21 | 36 | Male | Asian | Graduate | Sociology | 19 | 32 |
| 22 | 22 | Female | Asian | Graduate | Medicine | 7 | 12 |
| 23 | 19 | Female | Black | Undergraduate | Journalism | 10.5 | 18 |
| 24 | 18 | Female | Black | Undergraduate | Journalism/Communication | 7 | 13 |
| 25 | 19 | Female | Asian | Undergraduate | Psychology | 9 | 16 |
| 26 | 18 | Female | Asian | Undergraduate | Business | 10 | 16 |
| 27 | 18 | Male | Asian | Undergraduate | Biology | 6 | 14 |
