# Supplementary material for: Characteristics of Internet Addiction/Pathological Internet Use in U.S. University Students: A Qualitative-Method Investigation
Source: PLoS One. 2015 Feb 3;10(2):e0117372. doi: 10.1371/journal.pone.0117372 (PMC4315426; doi:10.1371/journal.pone.0117372)
Supplement: S2 Table — (DOCX) [file pone.0117372.s003.docx]

**Table S2** Data Set for Young’s Diagnostic Questionnaire (N = 27)

| Case ID | YDQ Score | Item1 | Item2 | Item3 | Item4 | Item5 | Item6 | Item7 | Item8 |
| --- | --- | --- | --- | --- | --- | --- | --- | --- | --- |
| 1 | 4 | 1 | 1 | 1 | 0 | 1 | 0 | 0 | 0 |
| 2 | 5 | 1 | 0 | 1 | 0 | 1 | 1 | 0 | 1 |
| 3 | 6 | 1 | 1 | 1 | 1 | 1 | 0 | 0 | 1 |
| 4 | 4 | 1 | 1 | 1 | 0 | 1 | 0 | 0 | 0 |
| 5 | 7 | 1 | 1 | 1 | 1 | 1 | 0 | 1 | 1 |
| 6 | 4 | 1 | 0 | 1 | 0 | 1 | 0 | 0 | 1 |
| 7 | 7 | 1 | 1 | 1 | 1 | 1 | 0 | 1 | 1 |
| 8 | 8 | 1 | 1 | 1 | 1 | 1 | 1 | 1 | 1 |
| 9 | 6 | 1 | 1 | 1 | 0 | 1 | 1 | 0 | 1 |
| 10 | 5 | 1 | 0 | 1 | 1 | 1 | 1 | 0 | 0 |
| 11 | 8 | 1 | 1 | 1 | 1 | 1 | 1 | 1 | 1 |
| 12 | 6 | 1 | 0 | 1 | 1 | 1 | 1 | 0 | 1 |
| 13 | 4 | 0 | 0 | 1 | 1 | 1 | 0 | 0 | 1 |
| 14 | 5 | 1 | 1 | 0 | 0 | 1 | 0 | 1 | 1 |
| 15 | 4 | 1 | 1 | 1 | 0 | 1 | 0 | 0 | 0 |
| 16 | 0 | 0 | 0 | 0 | 0 | 0 | 0 | 0 | 0 |
| 17 | 3 | 0 | 0 | 1 | 0 | 1 | 0 | 0 | 1 |
| 18 | 2 | 0 | 0 | 1 | 0 | 1 | 0 | 0 | 0 |
| 19 | 2 | 1 | 0 | 0 | 0 | 1 | 0 | 0 | 0 |
| 20 | 4 | 1 | 1 | 0 | 0 | 1 | 0 | 1 | 0 |
| 21 | 4 | 0 | 1 | 1 | 0 | 1 | 1 | 0 | 0 |
| 22 | 4 | 1 | 0 | 0 | 1 | 1 | 0 | 0 | 1 |
| 23 | 7 | 1 | 1 | 1 | 1 | 1 | 1 | 1 | 0 |
| 24 | 7 | 1 | 1 | 1 | 1 | 1 | 1 | 0 | 1 |
| 25 | 3 | 1 | 0 | 0 | 0 | 1 | 0 | 0 | 1 |
| 26 | 6 | 1 | 1 | 1 | 1 | 1 | 0 | 0 | 1 |
| 27 | 3 | 1 | 0 | 0 | 0 | 1 | 0 | 0 | 1 |
