# Supplementary material for: Characteristics of Internet Addiction/Pathological Internet Use in U.S. University Students: A Qualitative-Method Investigation
Source: PLoS One. 2015 Feb 3;10(2):e0117372. doi: 10.1371/journal.pone.0117372 (PMC4315426; doi:10.1371/journal.pone.0117372)
Supplement: S3 Table — (DOCX) [file pone.0117372.s004.docx]

**Table S3** Data set for the Compulsive Internet Use Scale (N = 27)

| Case ID | CIUS Score | Item1 | Item2 | Item3 | Item4 | Item5 | Item6 | Item7 | Item8 | Item9 | Item10 | Item11 | Item12 | Item13 | Item14 |
| --- | --- | --- | --- | --- | --- | --- | --- | --- | --- | --- | --- | --- | --- | --- | --- |
| 1 | 32 | 3 | 2 | 1 | 1 | 3 | 3 | 3 | 2 | 2 | 3 | 3 | 2 | 1 | 3 |
| 2 | 36 | 3 | 3 | 0 | 2 | 4 | 2 | 1 | 3 | 3 | 3 | 2 | 4 | 4 | 2 |
| 3 | 36 | 3 | 3 | 2 | 1 | 3 | 2 | 1 | 4 | 4 | 2 | 3 | 3 | 2 | 3 |
| 4 | 32 | 3 | 3 | 2 | 2 | 2 | 2 | 2 | 4 | 3 | 2 | 2 | 2 | 1 | 2 |
| 5 | 51 | 4 | 4 | 2 | 4 | 4 | 3 | 4 | 3 | 3 | 4 | 4 | 4 | 4 | 4 |
| 6 | 29 | 3 | 3 | 1 | 1 | 1 | 2 | 3 | 4 | 2 | 3 | 2 | 2 | 2 | 0 |
| 7 | 50 | 4 | 4 | 3 | 3 | 4 | 4 | 4 | 3 | 3 | 3 | 4 | 4 | 4 | 4 |
| 8 | 54 | 4 | 4 | 4 | 4 | 4 | 4 | 4 | 4 | 4 | 4 | 4 | 3 | 3 | 4 |
| 9 | 48 | 4 | 4 | 4 | 3 | 4 | 2 | 1 | 4 | 3 | 3 | 4 | 4 | 4 | 4 |
| 10 | 34 | 3 | 3 | 2 | 2 | 4 | 2 | 2 | 4 | 3 | 1 | 3 | 2 | 1 | 2 |
| 11 | 33 | 2 | 2 | 2 | 3 | 2 | 3 | 3 | 3 | 2 | 2 | 1 | 3 | 2 | 3 |
| 12 | 35 | 3 | 4 | 2 | 1 | 2 | 2 | 2 | 3 | 3 | 1 | 3 | 3 | 3 | 3 |
| 13 | 40 | 5 | 5 | 0 | 0 | 4 | 3 | 2 | 4 | 1 | 2 | 1 | 5 | 5 | 3 |
| 14 | 30 | 3 | 3 | 2 | 1 | 1 | 2 | 2 | 2 | 2 | 3 | 3 | 2 | 2 | 2 |
| 15 | 20 | 4 | 3 | 0 | 0 | 2 | 0 | 0 | 3 | 2 | 1 | 0 | 1 | 3 | 1 |
| 16 | 0 | 0 | 0 | 0 | 0 | 0 | 0 | 0 | 0 | 0 | 0 | 0 | 0 | 0 | 0 |
| 17 | 23 | 0 | 2 | 2 | 1 | 3 | 1 | 1 | 3 | 3 | 1 | 2 | 2 | 2 | 0 |
| 18 | 17 | 3 | 3 | 1 | 0 | 0 | 2 | 1 | 4 | 2 | 0 | 1 | 0 | 0 | 0 |
| 19 | 31 | 3 | 3 | 2 | 2 | 3 | 2 | 2 | 3 | 1 | 2 | 2 | 2 | 2 | 2 |
| 20 | 28 | 3 | 3 | 1 | 2 | 3 | 2 | 2 | 3 | 2 | 1 | 1 | 2 | 1 | 2 |
| 21 | 32 | 3 | 3 | 2 | 3 | 3 | 1 | 1 | 3 | 3 | 3 | 3 | 2 | 1 | 1 |
| 22 | 34 | 3 | 3 | 1 | 2 | 4 | 2 | 3 | 1 | 1 | 2 | 2 | 4 | 4 | 2 |
| 23 | 41 | 4 | 4 | 1 | 2 | 3 | 4 | 4 | 2 | 2 | 3 | 4 | 3 | 2 | 3 |
| 24 | 43 | 4 | 4 | 2 | 2 | 2 | 3 | 3 | 3 | 4 | 2 | 3 | 4 | 4 | 3 |
| 25 | 37 | 3 | 4 | 4 | 2 | 4 | 2 | 2 | 4 | 1 | 0 | 2 | 4 | 3 | 2 |
| 26 | 28 | 3 | 2 | 1 | 2 | 0 | 1 | 2 | 3 | 3 |  | 1 | 4 | 3 | 3 |
| 27 | 25 | 2 | 4 | 1 | 1 | 4 | 1 | 2 | 3 | 2 | 0 | 2 | 1 | 1 | 1 |
